# Manual Therapy Effects on Nonspecific Neck Pain Are Not Mediated by Mechanisms Related to Conditioned Pain Modulation: A Randomized Clinical Trial

**DOI:** 10.3390/jcm12123894

**Published:** 2023-06-07

**Authors:** Alberto Arribas-Romano, Josué Fernández-Carnero, Yeray González-Zamorano, Leonardo Rodríguez-Lagos, Isabel María Alguacil-Diego, Miguel Molina-Álvarez, David Morales Tejera, Francisco Mercado-Romero

**Affiliations:** 1 Escuela Internacional de Doctorado, Department of Physical Therapy, Occupational Therapy, Rehabilitation and Physical Medicine, Rey Juan Carlos University, 28922 Madrid, Spain; alberto.arribas@urjc.es (A.A.-R.); yeray.gonzalez@urjc.es (Y.G.-Z.); rodriguez.lirl@gmail.com (L.R.-L.); miguel.molina@urjc.es (M.M.-Á.); david.morales.tejera@urjc.es (D.M.T.); 2 Cognitive Neuroscience, Pain, and Rehabilitation Research Group (NECODOR), Faculty of Health Sciences, Rey Juan Carlos University, 28922 Madrid, Spain; francisco.mercado@urjc.es; 3 Department of Physical Therapy, Occupational Therapy, Rehabilitation and Physical Medicine, Rey Juan Carlos University, 28922 Madrid, Spain; isabel.alguacil@urjc.es; 4 Motion in Brains Research Group, Institute of Neuroscience and Sciences of the Movement (INCIMOV), Centro Superior de Estudios Universitarios La Salle, 28023 Madrid, Spain; 5 La Paz Hospital Institute for Health Research, IdiPAZ, 28029 Madrid, Spain; 6 Grupo de Investigación en Neurorrehabilitación del Daño Cerebral y los Trastornos del Movimiento (GINDAT), Facultad de Ciencias Experimentales, Universidad Francisco de Vitoria, 28223 Madrid, Spain; 7 Motion Analysis, Ergonomics, Biomechanics and Motor Control Laboratory (LAMBECOM), Department of Physical Therapy, Occupational Therapy, Rehabilitation and Physical Medicine, Faculty of Health Sciences, Rey Juan Carlos University, 28922 Madrid, Spain; 8 Area of Pharmacology, Nutrition and Bromatology, Department of Basic Health Sciences, Rey Juan Carlos University, Unidad Asociada I+D+i Instituto de Química Médica (IQM) CSIC-URJC, 28922 Madrid, Spain; 9 Area of Human Anatomy and Embriology, Department of Basic Health Sciences, Rey Juan Carlos University, 28922 Madrid, Spain; 10 Department of Physiotherapy, Human Physiology and Anatomy, Faculty of Physical Education and Physiotherapy, Vrije Universiteit Brussel, Laarbeeklaan 103, 1090 Brussels, Belgium; 11 Pain in Motion Research Group (PAIN), Department of Physiotherapy, Human Physiology and Anatomy, Faculty of Physical Education and Physiotherapy, Vrije Universiteit Brussel, 1090 Brussels, Belgium; 12 Department of Psychology, Faculty of Health Sciences, Rey Juan Carlos University, 28922 Madrid, Spain

**Keywords:** manual therapy, conditioned pain modulation, central pain mechanism, neck pain

## Abstract

Background. Manual therapy (MT) is a treatment recommended by clinical practice guidelines in the management of patients with neck pain. However, the mechanisms through which MT works remain unknown. The aim of the present study is to investigate if MT is mediated by mechanisms related to conditioned pain modulation (CPM), comparing the effects of painful with a pain-free MT treatment. Methods. A two-arm, parallel, randomized controlled clinical trial with concealed allocation and blinding of the outcome assessor was conducted in university students with chronic or recurrent nonspecific neck pain (NSNP). Participants received either a painful or pain-free MT session. Psychophysical variables including pressure pain thresholds, CPM, temporal summation of pain and cold pain intensity were assessed before and immediately after treatment. In addition, changes in neck pain intensity over the following 7 days and self-perceived improvement immediately and 7 days after treatment were assessed. Results: No significant differences were found between groups in any of the psychophysical variables or in patients’ self-perceived improvement. Only a significantly greater decrease in neck pain intensity immediately after treatment was found in the pain-free MT group compared to the painful MT group. Conclusion: The results suggest that the immediate and short-term effects of MT on NSNP are not mediated by CPM-related mechanisms.

## 1. Introduction

Neck pain is a prevalent condition that causes significant pain, disability, and financial burden [1,2]. Despite significant investment in research and treatment for neck pain, the age-standardized point prevalence, annual incidence, and years lived with disability due to neck pain have remained constant over the past three decades [2]. Furthermore, more than 20% of patients with acute neck pain continue to experience recurrent episodes or persistent pain [3]. Nonspecific neck pain (NSNP) is a common diagnosis for patients with neck pain who lack pathoanatomical causes or associated trauma that could explain their pain [4].

Manual therapy (MT) and exercise are the two most common used treatments by physiotherapists for patients with neck pain [5]. Clinical practice guidelines recommend integrating MT into the treatment protocol for neck pain patients [6,7,8]. However, the results of studies evaluating the effectiveness of MT in treating neck pain are very heterogeneous, making it difficult to draw clear conclusions [9,10,11,12,13]. It has been proposed that the individual variability in treatment response may be responsible for this inconclusive evidence [14]. Different phenotypic presentations of pain with varying responses of pain mechanisms to treatments can result in significant variation in effects between individuals [15]. Therefore, MT decision-making based on individual pain mechanisms would help to move toward precision medicine for managing neck pain. However, to reach this point, it is necessary to understand the pain mechanisms through which MT works [14].

It has been postulated that MT’s mechanical stimulation leads to neurophysiological responses in both the peripheral and central nervous system that are responsible for pain inhibition [14,16]. These responses could potentially explain the effects of MT on psychophysical test such as pain pressure thresholds (PPT), conditioned pain modulation (CPM) and temporal pain summation (TSP) suggested by meta-analyses [17,18]. Nevertheless, the specific mechanisms involved in these inhibitory responses remain unclear.

CPM is a paradigm used in humans to evaluate the “pain inhibits pain” effect. Specifically, it assesses the changes in perceived pain intensity or pain threshold with a noxious test stimulus applied heterotopically with a “conditioning” stimulus [19]. This is a proxy measure for assessing diffuse noxious inhibitory controls (DNIC). DNIC evaluates the inhibition of second-order wide dynamic range neurons by applying a noxious stimulus outside the receptive field of the recorded neuron [20,21]. However, the contribution of DNIC to the CPM paradigm remains unclear. Experts recommend using a sequential CPM protocol (reevaluating the test stimulus after the conditioning stimulus to avoid patient confusion) [22], and DNIC mechanisms are effective when both noxious stimuli are present. It is therefore unclear whether the inhibitory effects observed in sequential CPM assessments are due to residual DNIC effects or other mechanisms [23]. The same effect could potentially be caused by MT. In other words, the patient’s pain would be the first pain, and the second (conditioning) stimulus could be induced by some of the MT techniques. Therefore, the immediate and short-term effects of MT may be mediated by inhibitory mechanisms related to CPM. One study investigated this hypothesis in healthy participants by comparing the effects on psychophysical variables between pain-inducing versus pain-free manual pressure [24]. They found significant differences in the pressure pain threshold (PPT), thermal pain threshold and CPM between those who received pain-inducing manual pressure and those who received pain-free manual pressure. However, to our knowledge, no study has evaluated this hypothesis in a pain population treated with MT.

The inhibitory response generated by MT may depend on different aspects of individual patients such as the functional status of pronociceptive and antinociceptive mechanisms. Wilson et al. [24] found that baseline CPM was associated with greater changes in PPT in participants who received pain-inducing manual pressure. Another important aspect that has been shown to influence inhibitory responses are expectations [25,26,27]. These have been shown to be associated with the success of MT treatment in patients with neck pain [28].

Based on the information discussed above, the primary objective of the current study is to investigate if the effects of MT on patients with chronic or recurrent NSNP pain are mediated by CPM-related mechanisms. The study aimed to compare the effects of pain-inducing MT treatment versus pain-free MT treatment on PPTs, CPM, TSP and cold pain intensity. Additionally, the secondary objective was to evaluate any differences in the immediate and short-term (up to 7 days) effects on intensity of neck pain and patient self-perceived improvement. Finally, the study aimed to explore potential causes of variability in results by analyzing associations between baseline CPM, baseline TSP, expectations, and changes in PPTs, intensity of neck pain and self-perceived improvement in each treatment.

## 2. Materials and Methods

### 2.1. Design

A two-arm parallel randomized controlled clinical trial was conducted at the Faculty of Health Sciences of Rey Juan Carlos University. The study was approved by the Ethics Committee of Rey Juan Carlos University under the protocol number: ENM 35/22 2803202210022 and registered in ClinicalTrials.gov with ID: NCT05680688. Participants who met the inclusion criteria received an information sheet and were required to give informed consent. One of the physiotherapists in charge of the intervention (Y.G.-Z) generated a sequence of random numbers with an allocation ratio of 1:1 with GraphPad (www.ghaphpad.com). An external person concealed the allocation of patients in opaque envelopes numbered with the patients’ codes. The evaluator who was blinded to the allocation (A.A.-R) recruited the participants. The physiotherapists performing the intervention were the only persons authorized to open the envelope and they did so just before starting the treatment.

### 2.2. Participants

Participants were recruited through emails, posters on social media and University classroom presentations. The inclusion criteria were as follows: participants aged between 18 and 65 years, studying or interning at one of the universities in the community of Madrid (Spain), suffering from NSNP (pain in the neck region that is not attributable to a known specific cause such as herniated disc, myelopathy, fractures, spinal stenosis, neoplasm, etc., nor associated with traumatic events such as whiplash) that started more than three months ago and has persisted or has experienced two or more episodes with a mean pain score ≥ 2 on the Numeric Pain Rating Scale (NPRS) (0–10 points) [29] in the last week; and experienced pain on the day of measurement. Exclusion criteria were signs of radiculopathy or neuropathic pain, neck surgery, inflammatory rheumatic, neurological, cardiorespiratory, oncological, or psychiatric diseases, pregnancy and the inability to read and understand Spanish for filling out questionnaires.

### 2.3. Treatment

A physiotherapist specialized in manual therapy for musculoskeletal disorders performed one session of either painful or pain-free MT immediately after the baseline evaluation, based on group allocation. Patients in the painful MT group received a treatment that induced medium-intensity pain and were instructed to provide feedback every 15 s indicating a pain level of approximately 5 out of 10 points of pain on the NPRS, where 0 is no pain and 10 is the worst pain imaginable. If the pain level was not at 5, the therapist adjusted the intensity of the treatment accordingly. Patients in the pain-free MT group received pain-free treatment and had to report every 15 s whether the techniques were causing any pain (0 out of 10 on NPRS). If any pain was being caused, the therapist was responsible for reducing the intensity of the treatment.

The participants received thirty minutes of MT, which consisted of combined treatment of various MT techniques. This approach has been shown to have a greater effect on PPTs and pain reduction than using a single technique [30]. The following three techniques were performed in both groups, which allowed for quick and easy modification of treatment intensity to maintain the required pain intensity of the patient:

- **Posteroanterior passive joint mobilization:** The therapist placed the tip of their thumbs on the posterior surface of the spinous process, previously evaluated as the most painful, while gently resting the other fingers around the participant’s neck [31]. Oscillations were performed at a frequency of one oscillation per second for a total of nine minutes, divided into three sets of three minutes, with a one-minute rest interval.
- **Pressure:** The therapist applied digital ischemic compression on the point with the highest hyperalgesia [32] in each of the following regions: right upper trapezius, left upper trapezius, right paravertebral muscles and left paravertebral muscles. The pressure was maintained for one minute at each point.
- **Massage:** The therapist performed slow muscle fiber gliding techniques to control the pain being caused. The upper trapezius muscles were targeted for three minutes each, starting with the right side and then moving to the left, with passes from the acromion to the occipital region. Another three minutes were dedicated to the paravertebral musculature on each side, following the same order, with passes from the T1 vertebra to the occipital region.

During the entire intervention, participants in both groups remained in a relaxed position lying prone on the stretcher, with their cervical spine maintained in a neutral position.

### 2.4. Outcomes

All assessments were performed by an experienced physiotherapist in pain assessment for research studies who was blinded to the group allocation of each participant. Participants were instructed not to disclose their group allocation to the assessor during the assessment process. James [33] and Bang [34] blinding indexes were generated to assess whether or not satisfactory blinding of the assessor was maintained. For this purpose, at the end of the post-treatment evaluation, the evaluator indicated which treatment he believed the patient had received by indicating “painful MT”, “pain-free MT” or “don’t know”.

#### 2.4.1. Demographic and Clinical Characteristics

At baseline, demographic information including sex, age, weight, height, pain duration, and the mean and worse pain intensities during the last week were collected (NPRS). Additionally, the following questionnaires were used: Graded Chronic Pain Scale, to assess the severity of chronic pain [35,36,37], Central Sensitization Inventory (CSI) to identify symptoms related to central sensitization [38,39,40], Neck Disability Index to assess the level of disability perceived by the patient as a consequence of the neck pain [41,42], Pain Catastrophizing Scale (PCS) to assess catastrophizing cognitions and behaviors concerning pain [43], Tampa Scale for Kinesiophobia (TSK) to assess the degree of fear of movement and (re)injury [44,45], Beck Depression Inventory-*II* to assess depression [46,47,48,49], State Anxiety Inventory (STAI-S) to measure anxiety as a state [50,51,52], and Pain Anxiety Symptoms Scale (PASS-20) to assess pain anxiety [53,54].

#### 2.4.2. Psychophysical Variables

- *Pressure Pain Thresholds (PPT)*

PPTs were assessed at baseline and immediately after the intervention using a digital algometer (Model FPX, Wagner instruments, Greenwich, CT, USA). Local PPTs were assessed at the spinous process of C7 and bilaterally at the muscle belly of the trapezius muscle (midpoint between C7 and the acromion). Remote PPTs were evaluated bilaterally at the extensor carpi ulnaris and tibialis anterior. Participants were instructed to indicate when the pressure sensation became painful by saying “stop”. The algometer pressure for the evaluation was gradually increased at a rate of 1 kg/second. Data were collected in kg/cm^2^. The assessments of the points followed the order from top to bottom and right to left. This sequence was repeated, and the mean value of both measurements was calculated for each point. The mean between both sides at the bilateral points was used for the analysis. The reliability and reproducibility of the results obtained by algometry to measure the pressure pain threshold have been previously demonstrated, with an intraclass correlation coefficient of 0.84–0.96, indicating good to excellent reliability [55,56].

- *Conditioned pain modulation*

CPM has shown high test–retest reliability [57]. For the test stimulus, the PPT was measured at the nail bed of the thumb on the symptomatic or most painful side. For the conditioning stimulus, a sphygmomanometer was used. This was placed on the arm of the asymptomatic or less painful side, with its lower edge 3 cm proximal to the ulnar fossa. The cuff was inflated to 260 mmHg and held until the subject perceived pain of 6–7/10 on the NPRS [58]. The PPT was measured again, and cuff pressure was released. A final PPT measurement was performed to assess the sustained effect of CPM after 1 min [59]. Parallel CPM was calculated as the difference between the PPT during the conditioned stimulus minus the PPT before the conditioned stimulus. Sequential CPM was estimated as the difference between the PPT one minute after the conditioned stimulus minus the PPT before the conditioned stimulus.

- *Temporal summation of pain*

TSP is a noninvasive and indirect measure of central sensitization in humans, which refers to an increase in pain intensity with repetitive noxious stimuli. TS was elicited with 10 applications of the algometer at the individual PPT intensity perceived at the nail bed of the thumb on the asymptomatic or less painful side. The pressure was increased at a rate of approximately 2 kg/s to the previously determined PPT intensity [60]. Pulses were presented with an interstimulus interval of 1 s since this has previously shown to be optimal for inducing TS with pain pressure [61]. Before application of the first pressure pulse, subjects were instructed to rate the pain intensity of the first and 10th pulse on the NPRS. The TSP was calculated as the difference of the pain intensity of the 10th pulse minus the first pulse.

- *Cold pain intensity*

To evaluate cold pain intensity, participants were seated on a chair, and the ice application test was conducted on the anterior skin of their right forearm [62]. A cold pack was applied to the skin for 10 s, after which the participants were asked to rate the intensity of pain on NPRS. The procedure was repeated three times to obtain a mean value, with a 60 s rest period between measures to prevent TS of pain [63].

#### 2.4.3. Intensity of Neck Pain

Intensity of neck pain was evaluated with NPRS. The score is recorded on a Likert scale ranging from 0 (no pain) to 10 (worst pain imaginable). The NPRS has a moderately reliable ICC of 0.76 and a clinically important difference of 13% [64]. Neck pain intensity was assessed before and immediately after treatment. In addition, participants took a record sheet where they reported pain intensity at the following times: 4 h, after 1, 2, 3, 4, 5, 6 and 7 days.

#### 2.4.4. Self-Perceived Improvement by Global Rating of Change (GROC Scale)

The GROC scale assesses self-perceived improvement on a scale of 15 items, of which 7 are improvement and 7 are deterioration, and with 1 central item with no clinical change [65]. Clinically significant changes were determined based on values from the fourth item of improvement or deterioration, while values between the three improvement and three deterioration items were considered as no clinically significant changes [66]. The test–retest reliability has proven to be good (ICC 0.90) [67]. GROC was assessed immediately after treatment and in the short term (after 7 days), as noted in the record sheet.

#### 2.4.5. Expectations

To assess patient expectation, they were asked to indicate their agreement, disagreement, or uncertainty with the following statement regarding each treatment: “I believe that manual therapy intervention (painful or nonpainful) will significantly help to decrease my neck pain”. Positive expectations were classified as “agreeing” with the assigned treatment, while negative expectations were classified as “disagreeing” with the assigned treatment. Neutral expectations were classified as “uncertain”.

### 2.5. Statistical Analysis

Statistical analysis was conducted with SPSS software (IBM SPSS Statistics, version 28).

At baseline, descriptive statistics were used to summarize the characteristics and expectations of each group. Student’s *t*-tests, Wilconson’s W-test and Pearson’s chi^2^ -test were used to assess differences between the groups. To reduce the risk of dropout bias, analyses were conducted on an intention-to-treat basis.

To evaluate between-group differences in psychophysical variables, multiple linear regressions was performed using post-treatment outcome as the dependent variable, treatment (painful vs. pain-free MT) as the covariate of interest and baseline outcome as a supporting covariate.

Analysis of variance (ANOVA) with time (baseline, immediate post-treatment, 4 h and 1, 2, 3, 4, 5, 6 and 7 days) and group (painful vs. pain-free MT) was used to assess between-group differences in intensity of neck pain. The assumption of sphericity was checked with the Mauchly’s test and, in case of violation (*p* < 0.05), the Greenhouse-Geisser correction was used. The effect size was evaluated with partial eta squared considering 0.01 as small, 0.06 as moderate and 0.14 as large [68]. Post hoc contrasts were performed to assess intragroup effects at each time point with respect to the baseline. To assess differences between groups in the change at different time points with respect to baseline, an analysis of covariance (ANCOVA) was performed by introducing the baseline NPRS as a covariate in the model [69]. The level of statistical significance was set at *p* < 0.05 for most analyses, except for post hoc comparisons, where a more conservative threshold of *p* < 0.01 was used to correct for multiple testing.

To examine the association between baseline CPM, baseline TSP and expectations with the effect on PPTs and pain intensity of each treatment, a multiple regression model adjusted for baseline was employed. Additionally, to investigate the association of baseline CPM, baseline TSP and expectations with self-perceived improvement after each treatment, an ordered logistic regression model was used. A significance level of *p* < 0.01 was considered for both analyses, taking into account multiple comparisons.

### 2.6. Sample Size Calculation

The sample size was calculated to detect a greater than 30% change in the tibialis anterior (1.5 kg/cm^2^) [70] and greater than the minimum detectable change [71] with a standard deviation of 1.5 based on a previous study with a similar population [70]. A 1:1 intergroup ratio, a significance level of 0.05, 80% power and two-tailed, resulting in a requirement of 17 participants in each group. Estimating a loss ratio of 10%, 19 students per group were ultimately required. The STATA software (IC 16.1, StataCorp LLC, Lakeway Drive, College Station, TX, USA) was used for this calculation.

## 3. Results

**Figure 1:**
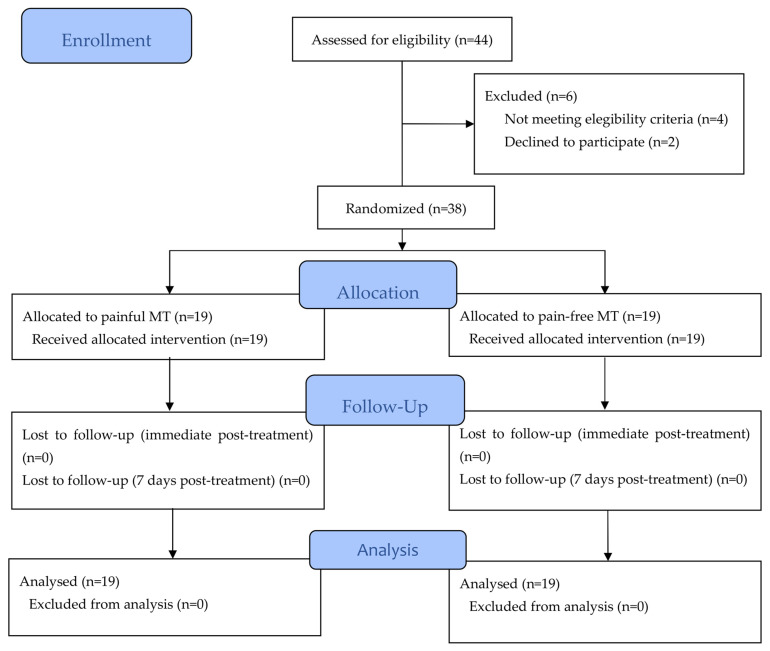
Flow diagram. MT: manual therapy.

A total of 38 students with NSNP were included in the trial and randomly assigned to either the painful MT group (n = 19) or the pain-free MT group (n = 19) (Figure 1).

The clinical and demographic characteristics and the expectations of each treatment group are presented in Table 1. Most participants were females (79%). Significant differences were only found between groups in terms of expectations of the effectiveness of the assigned treatment. More participants in the painful MT group had a positive expectation compared to the pain-free MT group. No adverse effects were reported in any of the patients. Slight reddening of the skin or momentary dizziness when moving from a lying to a sitting position encountered by some patients were not counted as they were not considered adverse effects.

### 3.1. Effects on Psychophysical Variables

Regressions adjusted for baseline revealed no differences between groups in any of the psychophysical variables (Table 2).

### 3.2. Effects on Intensity of Neck Pain

**Figure 2:**
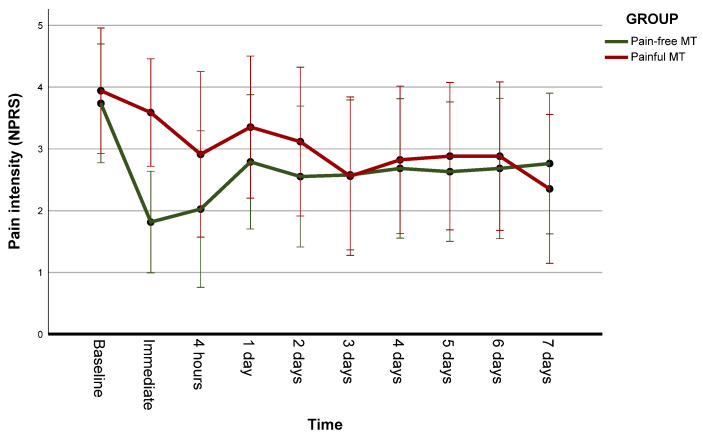
Effects on intensity of neck pain. The dots are the marginal means of each group at each time point and the vertical bars are the 99% confidence intervals. NPRS: numeric pain rating scale; MT: manual therapy.

The ANOVA results did not show significant group x time interactions (F = 1.86, *p* = 0.112, η^2^ = 0.052). However, there was a moderate effect of time (F = 3.32, *p* = 0.011, η^2^ = 0.087) (Table 3). The contrast tests revealed significant differences in the immediate post-treatment assessment and at 4 h compared to baseline in the pain-free MT group. In contrast, in the painful MT group, significant differences were found only at day 7. When comparing the effect between groups, there was a significantly greater decrease in post-treatment pain in the nonpainful MT group than in the painful MT group. However, no differences were found between groups at any other times (Table 3) (Figure 2).

### 3.3. Self-Perceived Improvement

There were no statistically significant differences between the two treatment groups in terms of self-perceived improvement immediately at post-treatment or at the 7-day follow-up. (Table 4).

### 3.4. Association of Baseline CPM and TSP with the Effects of Treatments on PPTs, Pain Intensity and Self-Perceived Improvement

No significant associations were found between baseline CPM or TSP and changes in PPTs, pain intensity or self-perceived improvement in either group (See Table S1 in Supplementary Materials).

### 3.5. Association of Expectations with the Effects of Treatments on PPTs, Pain Intensity and Self-Perceived Improvement

No significant associations were found between patient expectations and changes in PPTs, pain intensity or self-perceived improvement in either the painful or pain-free MT group (See Table S2 in Supplementary Materials).

### 3.6. Blinding Assessment

The evaluator forgot to indicate the treatment he thought the participant had received in 3 patients. See Table S3 in the Supplementary Materials for the distribution of assessor responses. James’ blinding index gave an estimate of 0.75 (95% CI: 0.60, 0.89), indicating that the study was well blinded. De Bang’s blinding index gave an estimate of 0.29 (95% CI: 0.07, 0.05) for the painful TM arm, and 0.00 (95% CI: −0.22, 0.22). These results can be directly interpreted as, for 29% of participants in the painful MT arm, the assessor correctly got their treatment right beyond chance, whereas this was 0% in the pain-free MT group.

## 4. Discussion

The purpose of this study was to investigate whether immediate or short-term MT effects could be related to CPM mechanisms. To achieve this goal, an MT that induced pain in patients was compared with a pain-free MT. The main findings indicated that there were no differences between both groups in the immediate effects on any of the psychophysical variables, nor in patients’ self-perceived improvement. However, in terms of pain intensity, participants who received pain-free MT experienced a significantly greater reduction immediately after the treatment compared to those who received painful MT. Nevertheless, there were no differences in pain intensity at any other time points. The pain-free MT group reported a significant decrease in pain intensity immediately post-treatment and at 4 h compared to baseline, whereas the painful MT group only reported significant differences on day 7.

The lack of differences in any of the psychophysical variables or patients’ self-perceived improvement between groups, except for pain intensity in favor of the pain-free MT group, suggests that CPM-related mechanisms may not mediate the effects of MT. Snodgrass et al. [31] also found no difference in PPTs between high-force and low-force neck mobilizations and a placebo in patients with neck pain. The absence of a significant increase in PPTs in the painful MT group, which was used as a proxy for the CPM paradigm, shortly after treatment is consistent with findings in animals, where DNIC-related mechanisms were only effective when two noxious stimuli were applied simultaneously [23]. The short time between the end of treatment by the physiotherapist and measurement of PPTs by the assessor in the present study may have been enough for the nociceptive inhibitory effect of the DNIC-related mechanisms to dissipate. Consequently, the results do not support the hypothesis that MT effects could be due to a residual effect of CPM and DNIC mechanisms. Nevertheless, these findings are not in line with the conclusions obtained by Wilson et al. [24] in healthy patients, where differences in PPTs between pain-inducing manual pressure and pain-free manual pressure were observed. These discrepancies in results between studies may be due to the lower efficacy of baseline CPM in many neck pain patients compared to healthy individuals [72]. Wilson et al. [24] found that healthy participants with an efficient baseline CPM who received pain-inducing manual pressure showed a greater increase in PPTs. Nonetheless, the present study found no association between baseline CPM or TSP and changes in PPTs, pain intensity or self-perceived improvement in either treatment. These within-group analyses should be interpreted cautiously since the sample size was not estimated for them. In contrast, a study in a population with lateral epicondylalgia found a significant correlation between CPM and manipulation-induced hypoalgesia [73]. Therefore, clinical prediction studies of MT are necessary to determine if CPM and TSP, as assessments of pain inhibition and facilitation capacity, can predict MT success.

An umbrella review [18] recently concluded that MT can increase PPTs immediately or in the short term in patients with chronic musculoskeletal pain. However, when considering the results obtained in terms of the different MT treatments and control groups used, the results were mixed. In the present study, none of the treatments increased PPTs in patients with neck pain, consistent with the findings of Snodgrass et al. [31] and Sterling et al. [74] in patients with neck pain and whiplash-associated disorders, respectively. These findings suggest that MT may not have hypoalgesic effects in patients with neck pain and that its effects on pain intensity and patients’ self-perceived improvement may be mediated by other mechanisms. However, in populations with knee osteoarthritis, end-range mobilization has been shown to produce immediate hypoalgesic effects, in opposition to not end-range mobilization and sham mobilization [75]. However, as the studies were based on the amount of movement generated by the mobilization and not on the intensity of the pain induced, it cannot be concluded that they are mediated by CPM-related mechanisms.

Patients who received pain-free MT reported greater immediate changes in neck pain intensity than those who received painful MT, which may be due to the pain induced in the latter group persisting after the session. Indeed, the painful MT group had no significant change in pain intensity until day 7, while the pain-free MT group had significant changes immediately after treatment and at 4 h. These results are in line with those reported by Snodgrass et al. [31] who found that the group receiving high-force mobilization reported more pain intensity immediately after treatment than the low-force group, but less at 4 days. The adaptation of the intensity of the treatments to the objectives of the study, which differs from clinical practice, may explain these findings. Inducing pain throughout the MT session is not common in normal clinical practice, which may have delayed the effects until days later in the painful MT group. Conversely, due to the low intensity of the treatment, effects may have only lasted a few hours in the pain-free MT group. However, both groups perceived andimprovement immediately after the session and no statistically significant difference was found between them. One possible explanation for this finding could be the significantly higher number of patients with positive expectations in the painful MT group compared to the pain-free MT group. However, no association between expectations and self-perceived improvement was observed in the painful MT group. In the study by Snodgrass et al. [31], 20 of the 21 participants in the high force mobilization group, despite increased pain after treatment, reported that they would be willing to undergo the treatment again if they attended physiotherapy. Bishop et al. [28] found that patients who had an expectation that manipulation would help that episode of neck pain and then received manipulation showed a higher probability of success. Management of expectations before and during treatment could have an important impact on patients’ satisfaction [76].

*Clinical Relevance*

The present study’s findings may help to move toward a mechanism-based approach to MT treatment. Specifically, it is indicated that MT’s immediate and short-term effects on pain in patients with neck pain are unlikely to be mediated by CPM-related mechanisms. This study shows clinicians that the decision to induce pain during their MT treatments should not be based on the idea that it generates hypoalgesia in patients. However, inducing pain produces an immediate self-perceived clinical improvement and a decrease in pain a few days after treatment, but the mechanisms involved are unknown.

*Limitations*

The present study has four main limitations that should be considered when interpreting the results. The main limitation is that the assessments in the hours and days following the intervention, as opposed to the immediate post-intervention, were self-reported unsupervised by the patient in a logbook. Therefore, there is a possibility of bias in the collection of these data.

Secondly, the sample size for the association analyses conducted in each treatment group was limited. Consequently, the conclusions drawn from these should be taken with caution.

Thirdly, assessor blinding was not successful in the painful MT group; they may have been conditioned in the evaluation of some participants.

Finally, the sample was composed of young university students in the field of health sciences in the community of Madrid, which limits the generalizability of the results to the general population of NSNP.

## 5. Conclusions

The results of this study indicate that the effects of MT on NSNP are not mediated by CPM-related mechanisms, as no differences were found in PPT changes between painful and pain-free MT. In addition, there were also no differences between groups in changes in CPM, TSP, and cold pain intensity. Patients who received pain-free MT reported a significantly greater decrease in pain intensity immediately after treatment compared to those who received painful MT. Painful MT did not show a statistically significant decrease in pain intensity until the seventh day measurement. Despite this, no differences were found in patients’ self-perceived improvement immediately after the session and at 7 days between the two groups.

## Figures and Tables

**Table 1:** Baseline demographic and clinical characteristics.

	Painful MT (n = 19)	Pain-Free MT (n = 19)	Statistic	Between Group *p*
**Sex**	15/4	15/4		
Female, *No. (%)*	15 (79)	15 (79)	Chi^2^ = 0.00	1.000
Male, *No. (%)*	4 (21)	4 (21)		
**Age (y)** ^b^	24 (20, 27)	23 (20, 27)	Z = 0.04	0.965
**Height (m)** ^a^	1.67 (0.08)	1.66 (0.11)	t = 0.54	0.590
**Weight (kg)** ^b^	62 (58, 70)	57 (54, 70)	Z = 1.20	0.231
**BMI (kg/m^2^)** ^b^	22.5 (20.2, 25.1)	21.7 (20.8, 23.2)	Z = 0.32	0.748
**Pain duration (mo)** ^b^	36 (36, 84)	36 (14, 72)	Z = 0.987	0.324
**Pain intensity (NPRS 0–10)**				
Current ^a^	3.84 (1.47)	3.74 (1.55)	t = 0.21	0.831
Mean in the last week ^a^	4.92 (1.36)	4.42 (1.36)	t = 0.18	0.860
Worst in the last week ^b^	6.5 (6, 8)	7 (5, 7)	Z = 0.19	0.846
**Graded Chronic Pain Scale** ^a^	26.7 (9.6)	31.1 (10.7)	t = −1.34	0.189
**Central Sensitization Inventory** ^a^	39.5 (10.9)	36.4 (11.6)	t = 0.85	0.401
**Neck Disability Index** ^a^	10.4 (3.3)	10.6 (4.9)	t = −0.19	0.847
**Pain Catastrophizing Scale (0–52)** ^b^	6 (3, 11)	9.89 (9.84)	Z = −0.19	0.849
**Tampa Scale for Kinesophobia (0–44)** ^a^	18.95 (4.2)	20.6 (5.33)	t = −1.08	0.286
**Pain Anxiety Symptoms Scale-20 (0–100)** ^a^	22.3 (13)	28.9 (17.1)	t = −1.35	0.187
**State Anxiety Inventory (0–60)** ^a^	24.6 (4.4)	24.3 (4.7)	t = 0.18	0.860
**Beck Depression Inventory-II (0–63)** ^a^	9.53 (5.34)	10.3 (9.15)	t = −0.32	0.748
**Expectations**				
Positive, *No. (%)*	17 (89%)	6 (32%)	Chi^2^ = 13.62	0.001 *
Neutral, *No. (%)*	1 (5%)	10 (53%)		
Negative, *No. (%)*	1 (5%)	3 (16%)		

* Significant differences (*p* < 0.05); ^a^ = Data were normally distributed in both groups and consequently: means and standard deviations are presented; ^b^ = The data were not normally distributed in any of the groups and consequently: medians and interquartile ranges are presented; CI: confidence interval; y = years; BMI: body mass index; mo = months; NPRS = numeric pain rating scale.

**Table 2:** Effects on psychophysical variables outcomes.

Variables	Painful MT (n = 19)			Pain-Free MT (n = 19)				
	Baseline Mean (SD)	Post-Treatment Mean (SD)	Diff. Intra-Group (Post-Base) Diff (95% CI)	Baseline Mean (SD)	Post-Treatment Mean (SD)	Diff. Intra-Group (Post-Base) Diff (95% CI)	Test	*p*-Value
PPT C7	3.09 (1.26)	3.03 (1.10)	−0.06 (−0.47; 0.35)	3.51 (1.44)	3.14 (1.14)	−0.37 (−0.89; 0.16)	t = 0.48	0.632
PPT trapezius	2.08 (0.77)	2.26 (0.74)	0.18 (−0.15; 0.51)	2.16 (0.91)	2.26 (0.97)	0.10 (−0.10; 0.31)	z = 0.32	0.749
PPT extensor ulnaris	4.77 (1.31)	4.49 (1.16)	−0.28 (−0.59; 0.25)	4.22 (1.38)	4.26 (1.39)	0.04 (−0.39; 0.46)	t = −0.85	0.403
PPT tibialis anterior	6.78 (2.55)	6.59 (1.95)	−0.18 (−0.81; 0.45)	6.29 (2.50)	6.16 (2.13)	−0.13 (−0.53; 0.27)	t = 0.26	0.793
Parallel CPM	0.98 (1.23)	0.43 (0.92)	−0.55 (−1.23; 0.14)	1.20 (0.92)	0.58 (1.42)	−0.62 (−1.30; 0.06)	t = −0.25	0.805
Sequential CPM	0.21 (0.99)	0.01 (0.77)	−0.20 (−0.83; 0.43)	0.49 (0.67)	0.14 (1.08)	−0.35 (−0.90; 0.20)	z = −0.31	0.760
TSP	3.5 (1.97)	3.58 (2.19)	0.08 (−0.73; 0.88)	2.5 (2.58)	3.03 (1.87)	0.53 (−0.77; 1.82)	t = 0.22	0.827
Cold pain intensity	4.74 (1.96)	4.44 (1.90)	−0.30 (−0.90; 0.31)	6.05 (1.78)	5.5 (1.93)	−0.55 (−1.17; 0.07)	t = −0.05	0.959

Significant differences (*p* < 0.05). MT: manual therapy; SD: standard deviation; Diff.: differences; CI: confidence interval; PPT: pressure pain threshold; CPM: conditioned pain modulation; TSP: temporal summation of pain.

**Table 3:** Effects on intensity of neck pain.

Follow-Up	Painful MT (n = 19)		Pain-Free MT (n = 19)		Group Diff. (Adj. by Baseline) Mean Diff. (99% CI)
	Mean (SD)	Diff. with Baseline Mean Diff. (99% CI)	Mean (SD)	Diff. with Baseline Mean Diff. (99% CI)	
**Baseline**	3.94 (1.52)		3.74 (1.55)		
**Post-treatment**	3.59 (1.50)	−0.35 (−1.58, 0.87)	1.82 (1.12)	−1.92 (−3.08, −0.77) *	1.74 (0.54, 2.95) *
**4 h**	2.91 (2.40)	−1.03 (−2.54, 0.48)	2.03 (1.62)	−1.71 (−3.13, −0.29) *	0.83 (−1.01, 2.67)
**1 day**	3.35 (1.73)	−0.59 (−1.90, 0.72)	2.79 (1.74)	−0.95 (−2.19, 0.29)	0.50 (−1.05, 2.05)
**2 days**	3.12 (1.68)	−0.82 (−2.14, 0.49)	2.55 (1.94)	−1.18 (−2.43, 0.06)	0.49 (−1.12, 2.10)
**3 days**	2.56 (1.68)	−1.38 (−2.78, 0.01)	2.58 (2.14)	−1.16 (−2.48, 0.16)	−0.09 (−1.82, 1.63)
**4 days**	2.82 (1.67)	−1.12 (−2.38, 0.142)	2.68 (1.92)	−1.05 (−2.24, 0.14)	0.05 (−1.51, 1.62)
**5 days**	2.88 (1.65)	−1.06 (−2.42, 0.30)	2.63 (1.93)	−1.11 (−2.39, 0.18)	0.19 (−1.43, 1.81)
**6 days**	2.88 (1.87)	−1.06 (−2.40, 0.29)	2.68 (1.76)	−1.05 (−2.33, 0.22)	0.13 (−1.49, 1.75)
**7 days**	2.35 (1.94)	−1.59 (−2.73, −0.45) *	2.64 (1.66)	−0.97 (−2.05, 0.11)	−0.53 (−2.01, 0.95)

* Significant differences (*p* < 0.01). MT: manual therapy; Diff.: differences; CI: confidence interval; SD: standard deviation; Adj.: adjusted.

**Table 4:** Self-perceived improvement.

	Painful MT (n = 19) Median (IQR)	Pain-Free MT (n = 19) Median (IQR)	Difference (95% CI)	Between Group *p*
**GROC post-treatment**	4 (2–5)	3 (1–5)	1 (−1, 2)	0.308
**GROC post-7 days**	3 (1–4)	1 (0–3)	1 (0, 3)	0.149

Significant differences (*p* < 0.05). MT: manual therapy; IQR: Interquartile range; CI: confidence interval; GROC: global rating of change.

## Data Availability

The data presented in this study are available on request from the corresponding author. The data are not publicly available because the privacy of research participants could be compromised.

## Supplementary Materials

The following supporting information can be downloaded at: https://www.mdpi.com/article/10.3390/jcm12123894/s1, Table S1: Association of baseline CPM and TSP with the effects of treatments on pain intensity and self-perceived improvement. Table S2: Association of expectations, pain catastrophism and pain anxiety with the effects of treatments on pain intensity and self-perceived improvement. Table S3: Number of subjects by treatment assignment and assessor’s guess.
