# Supplementary material for: Manual Therapy Effects on Nonspecific Neck Pain Are Not Mediated by Mechanisms Related to Conditioned Pain Modulation: A Randomized Clinical Trial
Source: J Clin Med. 2023 Jun 7;12(12):3894. doi: 10.3390/jcm12123894 (PMC10298868; doi:10.3390/jcm12123894)
Supplement: Supplementary file 1 [file jcm-12-03894-s001.zip › jcm-2413958-supplementary.pdf]

Supplementary Materials

Table S1. Association of baseline CPM and TSP with the effects of treatments on pain intensity and self-perceived improvement.

	Painful MT (n = 19)			Pain-free MT (n = 19)		
	**CPM parallel**	**CPM sequential**	**TSP**	**CPM parallel**	**CPM sequential**	**TSP**
**PPT C7**, t statistic (p value)	0.94 (0.363)	1.36 (0.195)	-0.55 (0.587)	-0.26 (0.800)	-0.95 (0.357)	-0.00 (0.996)
**PPT trapezius**, t statistic (p value)	1.03 (0.320)	2.48 (0.025)	-0.35 (0.730)	0.22 (0.826)	-0.87 (0.395)	0.70 (0.495)
**PPT extensor ulnaris**, t statistic (p value)	0.19 (0.853)	0.61 (0.551)	-0.38 (0.710)	0.32 (0.753)	-0.37 (0.718)	-0.45 (0.660)
**PPT tibialis anterior**, t statistic (p value)	0.93 (0.368)	0.144 (0.170)	-0.56 (0.581)	0.14 (0.894)	-1.45 (0.167)	0.20 (0.894)
**Pain intensity post-treatment**, t statistic (p value)	-0.69 (0.499)	0.88 (0.393)	-0.43 (0.674)	0.85 (0.408)	1.59 (0.131)	0.96 (0.352)
**Pain intensity post-7 days**, t statistic (p value)	-0.70 (0.498)	-0.89 (0.392)	-0.78 (0.449)	-2.61 (0.019)	-0.14 (0.892)	-0.07 (0.944)
**GROC post-treatment**, z statistic (p value)	-0.85 (0.396)	-0.37 (0.714)	-0.28 (0.779)	-0.81 (0.416)	-0.74 (0.456)	-0.39 (0.697)
**GROC post-7 days**, z statistic (p value)	-2.23 (0.026)	0.44 (0.659)	1.48 (0.140)	1.08 (0.278)	0.38 (0.707)	0.46 (0.648)

* Significant differences (p < 0.01). MT: manual therapy; CPM: conditioned pain modulation; TSP: temporal summation of pain: PPT: pressure pain threshold; GROC: global rating of change

Table S2. Association of expectations, pain catastrophism and pain anxiety with the effects of treatments on pain intensity and self-perceived improvement.

	**Painful MT (n = 19)**	**Pain-free MT (n = 19)**
	**Expectations**	**Expectations**
**PPT C7,** F statistic (p value)	0.35 (0.709)	2.40 (0.125)
**PPT trapezius,** F statistic (p value)	0.10 (0.902)	5.43 (0.017)
**PPT extensor ulnaris,** F statistic (p value)	0.29 (0.751)	0.64 (0.541)
**PPT tibialis anterior,** F statistic (p value)	0.97 (0.400)	0.52 (0.602)
**Pain intensity post-treatment,** F statistic (p value)	0.31 (0.738)	0.65 (0.537)
**Pain intensity post-7, days,** F statistic (p value)	6.15 (0.013)	0.55 (0.591)
**GROC post-treatment,** Chi^2 (p value)	1.06 (0.588)	1.76 (0.414)
**GROC post-7 days,** Chi^2 (p value)	0.75 (0.687)	0.21 (0.901)

* Significant differences (p < 0.01). MT: manual therapy; PPT: pressure pain threshold; GROC: global rating of change

Table S3. Number of subjects by treatment assignment and assessor's guess

**Assignment**	**Assessor's response**			
	**Painful MT**	**Pain-free MT**	**Don't know**	**Total**
**Painful MT**	6	1	10	17
**Pain-free MT**	3	3	12	18
**Total**	9	4	22	35

MT: manual therapy
